# The Mobile App Development and Assessment Guide (MAG): Delphi-Based Validity Study

**DOI:** 10.2196/17760

**Published:** 2020-07-31

**Authors:** Pere Llorens-Vernet, Jordi Miró

**Affiliations:** 1 Unit for the Study and Treatment of Pain-ALGOS Research Center for Behavior Assessment Universitat Rovira i Virgili Tarragona Spain

**Keywords:** assessment, Delphi method, MAG, mobile apps, mobile health, validity, guide

## Abstract

**Background:**

In recent years, there has been an exponential growth of mobile health (mHealth)–related apps. This has occurred in a somewhat unsupervised manner. Therefore, having a set of criteria that could be used by all stakeholders to guide the development process and the assessment of the quality of the apps is of most importance.

**Objective:**

The aim of this paper is to study the validity of the Mobile App Development and Assessment Guide (MAG), a guide recently created to help stakeholders develop and assess mobile health apps.

**Methods:**

To conduct a validation process of the MAG, we used the Delphi method to reach a consensus among participating stakeholders. We identified 158 potential participants: 45 patients as potential end users, 41 health care professionals, and 72 developers. We sent participants an online survey and asked them to rate how important they considered each item in the guide to be on a scale from 0 to 10. Two rounds were enough to reach consensus.

**Results:**

In the first round, almost one-third (n=42) of those invited participated, and half of those (n=24) also participated in the second round. Most items in the guide were found to be important to a quality mHealth-related app; a total of 48 criteria were established as important. “Privacy,” “security,” and “usability” were the categories that included most of the important criteria.

**Conclusions:**

The data supports the validity of the MAG. In addition, the findings identified the criteria that stakeholders consider to be most important. The MAG will help advance the field by providing developers, health care professionals, and end users with a valid guide so that they can develop and identify mHealth-related apps that are of quality.

## Introduction

Mobile apps are increasingly being used for health care [1-3]. The implementation of mobile devices such as phones, patient monitoring devices or personal digital assistants, and wireless devices has proven that they can be used for improving communication between patients and health professionals [4], and improving adherence to treatment [5]. Importantly, recent reports have suggested that smartphones have become the most popular technology among physicians [6,7]. In addition, there has been a sharp increase in the use of these technologies by the general population. For example, official estimates indicate that in 2019 a total of 65% of people had a smartphone, and by 2025, this figure will have increased to 80% [8].

However, this increase in use has occurred in a somewhat unsupervised manner; that is to say, it has not been regulated or supervised in any way. In addition, a large number of mobile health (mHealth) apps have been developed without any rigorous scientific basis [9,10] or having undergone any validation process, thus undermining the confidence of both patients and health care professionals [11]. Moreover, information privacy practices are not transparent to users and, in many cases, are absent, opaque, or irrelevant [12]. Finally, there is mounting evidence to show that this lack of control and development without guidance is placing consumers at risk [13].

In an attempt to solve this problem, and guarantee the quality of existing and future health apps, various government-related initiatives have been taken at the regional level (eg, the proposal “AppSalut” [14,15] in Catalonia and the “AppSaludable Quality Seal” [16] in Andalusia, Spain), the national level (eg, “Good practice guidelines on health apps and smart devices [mobile health or mhealth]” [17] in France; “Health apps & co: safe digital care products with clearer regulations” [18] in Germany; “Medical devices: software applications [apps]” [19] in the United Kingdom; “Policy for Device Software Functions and Mobile Medical Applications” [20] in the United States; “Regulation of Software as a Medical Device” [21,22] in Australia), and the international level, such as the “Green Paper on mobile health” by the European Commission [23]. In general, these initiatives provide recommendations and regulations to establish how health apps should be and guarantee their quality. However, they show important differences on the key criteria. For example, “Appsalut” emphasizes usability issues [14,15], while “Regulation of Software as a Medical Device” emphasizes safety [21,22] as it equates health apps with medical devices. Clinicians and researchers have also attempted to provide specific solutions to this major problem [24]. For example, Stoyanov and colleagues [25] developed a scale to classify and rate the quality of mHealth apps. There have also been other attempts to provide alternatives for assessing mHealth apps (eg, [26,27]), each one of which suggests its own quality criteria. All these attempts have positive and negative characteristics. A major limitation common to many of these initiatives is that they have been created from one narrow perspective and focusing on, for example, a specific health problem or intervention such as emergency interventions [27] or a stakeholder such as adolescents [26]. In addition, some of them have been created from a specific perspective, for example, taking into account usability issues rather than safety. Thus, there is no common set of criteria that can be used by all stakeholders to guide the development process and the assessment of the apps’ quality.

Recently, to help overcome these limitations, we conducted a study to develop such a guide: the Mobile App Development and Assessment Guide (MAG) [28]. We studied the guidelines, frameworks, and standards available in the field of health app development, with a particular focus on the world regions where the mHealth market was most significant, and pinpointed the criteria that could be recommended as a general standard. We suggested a guide containing 36 criteria that could be used by stakeholders. Our preliminary study showed that stakeholders found them to be important. They also found them easy to understand and use [28].

However, that study had some limitations. Most importantly, although the criteria identified underwent a preliminary analysis of comprehensibility and importance by a selected group of stakeholders (ie, health care experts, engineers, and potential patients), they did not undergo a validation process. Therefore, to address this issue, here we use the Delphi method [29,30] to analyze the validity of the MAG. By using this method, we also want to explore whether new criteria could be included to improve the guide. We also want to examine the importance of these criteria as perceived by the stakeholders.

## Methods

### Procedure

The Delphi method was created for people to reach consensus by answering questions in an iterative process [29]. Although the traditional Delphi process has an open initial phase [29], in this study we use a modified Delphi process, which provides a common starting point for discussion. This modified Delphi method is widely used, as it saves time and does not interfere with the original tenets of the method that participants can give suggestions and inputs at any stage [31]. It has been shown that results from Delphi-based studies offer more accurate answers to difficult questions than other prognostication techniques [32]. This modified Delphi method and the judgment of people are acknowledged as legitimate and valid inputs to generate forecasts, and have been used in many different areas to reach consensus on such strategic issues as the identification of health care quality indicators [33]; predictors of chronic pain and disability in children [34]; predictors of chronic pain in adults with cancer [35]; the needs of adolescents and young adult cancer survivors [36]; and, in the mHealth field, to develop an assessment framework for electronic mental health apps [37].

### Participants

Our goal was to recruit 30 stakeholders, as this number has been shown to be sufficient for this kind of study [38,39], from any of the following groups: (1) health care professionals, (2) developers of health-related apps, and (3) users of health apps.

To identify potential participants and ensure an appropriate panel of stakeholders, we adopted five strategies: (1) we searched for national (Spain) and international organizations or associations of digital health professionals to make contact with health professionals knowledgeable about the topic; (2) we searched for medical health apps in the app stores of the main smartphone systems (Android and iOS), identified the most downloaded and best rated apps, and searched for their developers to ensure the participation of experienced individuals; (3) we searched for national (Spain) and international organizations to recruit patients with experience in the use of health-related apps or with an interest in this area; (4) we made a general call through the social networks of our research group to increase the likelihood of recruiting participants who satisfied the inclusion requirements; and (5) we asked researchers and clinicians who we personally knew were experts in the field to participate and help us identify other potential participants.

We identified 158 potential participants from Europe, Asia, Australia, and North and South America. They were multidisciplinary and included health care professionals, patients as potential end users, and developers.

### Survey Content and Procedure

We developed a list of items on the basis of the criteria in the MAG [28]. Some of the criteria were broad and encompassed several issues and characteristics, so we broke them down into specific items to facilitate the comprehensibility and accuracy of responses. For example, the original criterion “The app can be consulted in more than one language. All languages adapt appropriately to the content interface” was divided into two items: “The app can be consulted in more than one language” and “All languages adapt appropriately to the content interface.”

When the set of items was ready, it was moved to an online survey so that it could be distributed to participants more easily. Potential participants received an email with explanations about the study and a link to the survey. All the information was available in Spanish and English.

The survey included some sociodemographic questions and 56 items for rating, which were grouped in the same categories as the original guide, such as usability [28]. On a numerical rating scale from 0 (not important at all) to 10 (extremely important), participants had to report how important they considered each one to be for the quality of a health-related mobile app. Participants were also given instructions to include any other item they felt was important and missing from the original list. Like the original items, these new items were also rated. Participants were informed that only the criteria that received a score of 9 or higher from at least 75% of the participants would be included in the final list of criteria that a health-related app should meet. The rest were discarded.

Study data were collected and managed using LimeSurvey tools (LimeSurvey GmbH) [40]. We computed means and standard deviations of the demographics to describe the sample of participants. We used paired *t* tests (two-tailed) to study potential differences in the variance of the items between rounds and of the potential changes in the age or sex of participants. To study the consensus, mean, standard deviation, 95% confidence interval (with the lower and upper values for each item), and significance level (*P*<.05) were computed. All data analyses were performed using SPSS v.25 for Windows (IBM Corp).

### Delphi Rounds

In the first round, the survey was sent to 158 potential participants: 45 patients as potential end users, 41 health care professionals, and 72 developers. They were informed about the study and the requirements to participate. Participants were given 3 weeks to respond, during which time a reminder was sent each week to maximize the involvement of as many participants as possible. The survey took approximately 15 minutes. The answers were analyzed, and some new items were added in response to the suggestions of the participants.

In the second round, the results of the first iteration were sent by email to all the participants who had provided responses to the initial survey so that they could see their position and the position of the group as a whole on the items, as well as the level of agreement among the participants. This information was given so that participants in the group could re-examine their initial responses, in light of the group’s opinion. Participants in this second round were asked to respond to the revised survey. Again, they were given 3 weeks to answer. The Delphi methodology requires that this procedure be repeated until participants’ responses reach stability or when a point of diminishing returns is reached [39].

**Figure 1:**
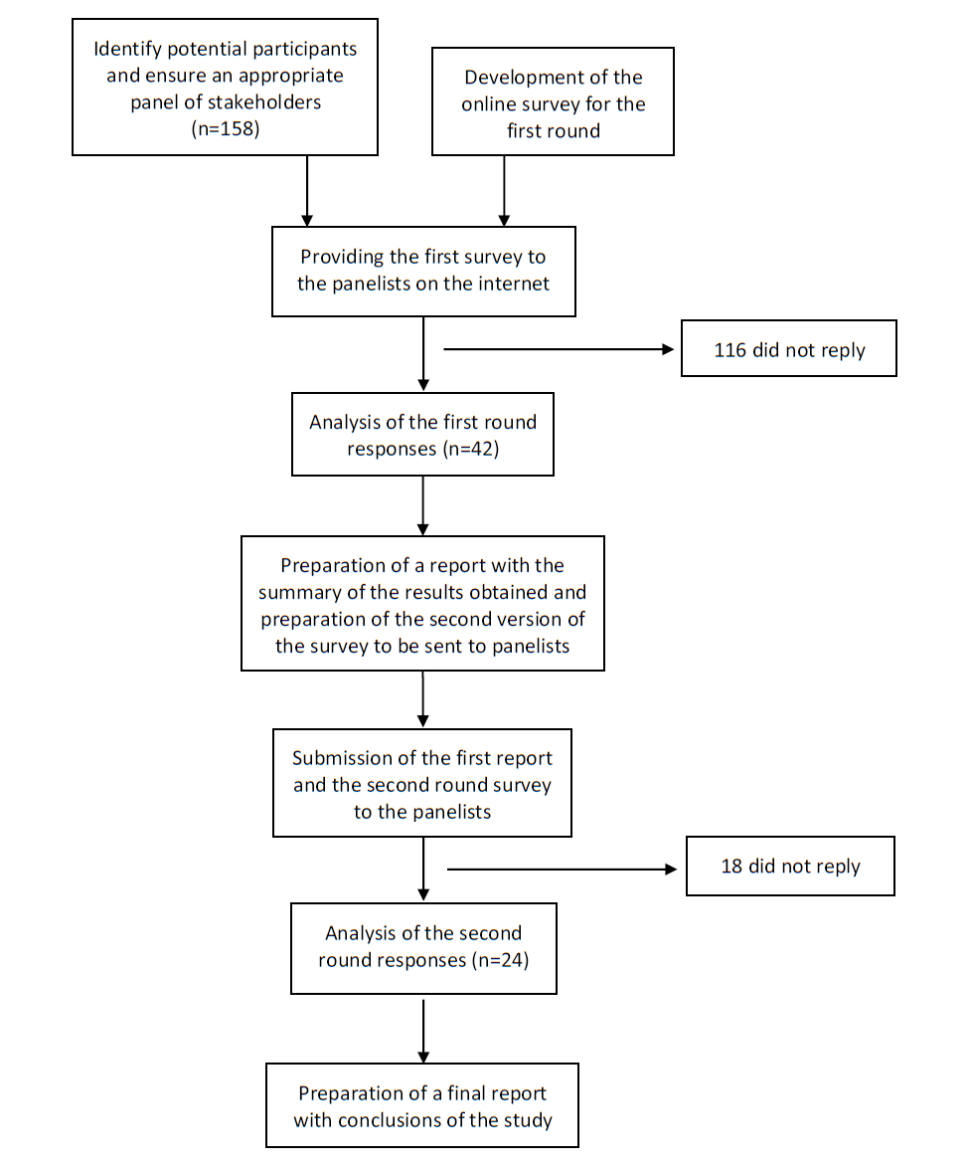
Steps in the Delphi poll.

The stability of responses was the criterion used to identify that consensus had been reached on any given question [30,38]. In this study, stability was reached after two rounds (see the Results section), which is consistent with the findings of previous Delphi studies (eg, [35,41]). We considered that consensus was reached on a particular criterion when 75% of the participants rated it with at least a 9 [34]. If a criterion was rated with a 9 or more by at least 90% of the participants, we considered it to be of key importance for an mHealth-related app. The results only showed statistically significant differences for two items (see the Results section). Thus, given the stability of the responses, we decided to stop the iteration process after round 2. Figure 1 describes the steps of the study.

## Results

### Round 1

Of the 158 stakeholders invited, 42 (27%) responded to the first round. The demographic characteristics of the participants in each round are shown in Table 1. There were no statistically significant differences in terms of sex or age between those who were invited and those who participated. Only a small increase in the mean age of participants and female participation was detected between rounds (see Table 1).

Multimedia Appendix 1 summarizes all the information about participants’ responses to the initial 56 items.

To determine consensus on the items, we examined the percentage of participants who agreed on their importance. Items with an agreement ≥75% of participants were considered to have reached consensus. We also used confidence intervals, instead of discrete estimation, because they have less error (see [34] for a similar procedure). Out of the total 56 items, participants reached consensus on the importance of 32 (57%) of the items (ie, at least 75% of the participating stakeholders rated their importance with a 9 or higher on the 0-10 scale).

In this first round, participants added 36 new items to the original list. As previously described, in response to participants’ suggestions, changes were made to items 3, 51, 68, and 73. In addition, items 33 and 69 were divided in two, as several participants considered that they included two different clauses (see Multimedia Appendix 1).

**Table 1:** Sample characteristics in each round.

Characteristics		Invited participants (N=158)	Participants round 1 (n=42)	Participants round 2 (n=24)
**Sex, n (%)^a^**				
	Male	17 (42.5)	15 (44.12)	9 (37.5)
	Female	23 (57.5)	19 (55.88)	15 (62.5)
**Stakeholder group, n (%)**				
	Health care professionals	45 (28.48)	16 (38.1)	9 (37.5)
	Developers	41 (25.95)	14 (33.33)	8 (33.33)
	Final users	72 (45.57)	12 (28.57)	7 (29.17)
Age (years), mean^a^		39.82	41.39	43.08
**Citizenship, n (%)^a^**				
	Spain	78 (49.37)	28 (82.35)	20 (83.33)
	United States	13 (8.23)	1 (2.94)	1 (4.17)
	Argentina	1 (0.63)	1 (2.94)	1 (4.17)
	Italy	1 (0.63)	1 (2.94)	1 (4.17)
	Australia	6 (3.8)	1 (2.94)	1 (4.17)
	United Kingdom	15 (9.49)	2 (5.88)	0 (0.00)
	Canada	8 (5.06)	0 (0.00)	0 (0.00)
	Brazil	3 (1.9)	0 (0.00)	0 (0.00)
	India	6 (3.8)	0 (0.00)	0 (0.00)
	Indonesia	1 (0.63)	0 (0.00)	0 (0.00)
	Germany	5 (3.16)	0 (0.00)	0 (0.00)
	Poland	2 (1.27)	0 (0.00)	0 (0.00)
	Ireland	2 (1.27)	0 (0.00)	0 (0.00)
	Turkey	1 (0.63)	0 (0.00)	0 (0.00)
	Kazakhstan	1 (0.63)	0 (0.00)	0 (0.00)
	Switzerland	3 (1.9)	0 (0.00)	0 (0.00)
	Colombia	1 (0.63)	0 (0.00)	0 (0.00)
	Romania	1 (0.63)	0 (0.00)	0 (0.00)
	Hungary	1 (0.63)	0 (0.00)	0 (0.00)
	Lithuania	1 (0.63)	0 (0.00)	0 (0.00)
	Bulgaria	2 (1.27)	0 (0.00)	0 (0.00)
	Greece	1 (0.63)	0 (0.00)	0 (0.00)
	Sweden	2 (1.27)	0 (0.00)	0 (0.00)
	Israel	1 (0.63)	0 (0.00)	0 (0.00)
	Belgium	2 (1.27)	0 (0.00)	0 (0.00)

^a^Information was not available for all participants.

### Round 2

Of the 42 that participated in the first round, 24 (57%) of the stakeholders participated in the second round. Out of the total 92 items, a total of 48 items (52%) were rated with a 9 or more by at least 75% of the participants (see Table 2 below).

The consensus on the importance of the 32 items in round 1 was maintained in round 2, except for item 69, which fell below the criteria of 75% agreement. On the other hand, items 8, 32, 46, and 47 did not reach consensus in round 1 but did in round 2. Consensus was also reached on the importance of 14 of the new items suggested by participants. Of all the items, 9 were particularly important (ie, at least 90% of participants rated their importance with a 9 or higher).

By categories, the number of items for which consensus was reached were *usability*: 8 of 18 items (44% of the total in the category); *privacy*: 14 of 19 items (74%); *security*: 9 of 13 items (69%); *appropriateness and suitability*: 2 of 5 items (40%); *transparency and content*: 2 of 11 items (18%); *safety*: 7 of 8 items (88%), *technical support and updates*: 2 of 9 items (22%); and *technology*: 4 of 9 items (44%).

**Table 2:** Items that reached consensus about their importance.

Category and item		Round 1 (n=42)			Round 2 (n=24)		
		Consensus, n (%)	Mean (SD)	95% CI	Consensus, n (%)	Mean (SD)	95% CI
**Usability**							
	1. The app has been tested by potential users before being made available to the public.	33 (78.57)	9.14 (1.92)	8.56-9.72	20 (83.33)	9.21 (1.61)	8.56-9.85
	2. It is easy to use (ie, navigation is intuitive).	39 (92.86)	9.67 (0.61)	9.48-9.85	21 (87.50)	9.50 (0.72)	9.21-9.79
	3. Functionality is adapted to the purpose of the app.	40 (95.24)	9.74 (0.63)	9.55-9.93	21 (87.50)	9.42 (0.93)	9.05-9.79
	4. Functionality is adjusted according to the profile and needs of the targeted user.	—^a^	—	—	19 (79.17)	9.08 (0.93)	8.71-9.45
	5. Access is adapted for people with disabilities.	—	—	—	19 (79.17)	9.00 (1.41)	8.43-9.57
	6. It complies with regulatory accessibility standards.	—	—	—	18 (75.00)	9.17 (1.13)	8.71-9.62
	7. The language used makes it accessible to any user.	—	—	—	19 (79.17)	9.04 (1.55)	8.42-9.66
	8. All users have access to all resources regardless of their capabilities.	28 (66.67)	8.60 (1.67)	8.09-9.10	18 (75.00)	9.08 (1.06)	8.66-9.51
**Privacy**							
	9. The app gives information about the terms and conditions of purchases in the app.	35 (83.33)	9.29 (1.90)	8.71-9.86	21 (87.50)	9.50 (1.18)	9.03-9.97
	10. It must only ask for user data that is essential for the app to operate.	34 (80.95)	9.26 (1.53)	8.80-9.72	18 (75.00)	8.92 (2.04)	8.10-9.73
	11. It gives information about access policies and data processing, and ensures the right of access to recorded information.	34 (80.95)	9.02 (2.25)	8.34-9.70	18 (75.00)	9.38 (0.97)	8.99-9.76
	12. It gives information about possible commercial agreements with third parties.	32 (76.19)	8.79 (2.54)	8.02-9.55	20 (83.33)	9.17 (2.12)	8.32-10.01
	13. It clearly allows the user the option of nontransfer of data to third parties or for commercial purposes.	—	—	—	23 (95.83)	9.71 (0.55)	9.49-9.93
	14. It guarantees the privacy of the information recorded.	39 (92.86)	9.71 (0.77)	9.48-9.95	20 (83.33)	9.46 (1.10)	9.02-9.90
	15. It requires users to give their express consent.	36 (85.71)	9.12 (2.19)	8.46-9.78	19 (79.17)	9.38 (1.01)	8.97-9.78
	16. It warns of the risks of using the app.	36 (85.71)	9.33 (1.86)	8.77-9.89	19 (79.17)	9.25 (1.19)	8.77-9.73
	17. It tells users when it accesses other resources on the mobile device such as their accounts or their social network profiles.	36 (85.71)	9.33 (1.76)	8.80-9.87	22 (91.67)	9.71 (0.75)	9.41-10.01
	18. It takes measures to protect minors in accordance with current legislation.	38 (90.48)	9.43 (1.74)	8.90-9.96	23 (95.83)	9.79 (0.51)	9.59-10.00
	19. Confidential user data is protected and anonymized, and there is a privacy mechanism so that users can control their data.	38 (90.48)	9.60 (1.06)	9.27-9.92	21 (87.50)	9.46 (1.18)	8.99-9.93
	20. It offers to erase the data when the service is finished.	—	—	—	19 (79.17)	9.04 (1.68)	8.37-9.71
	21. It gives information about privacy policies in a simple and understandable way.	—	—	—	20 (83.33)	9.33 (1.09)	8.90-9.77
	22. It complies with all current privacy laws.	—	—	—	22 (91.67)	9.54 (1.28)	9.03-10.06
**Security**							
	23. The app has encryption mechanisms for storing, collecting, and exchanging information.	35 (83.33)	9.40 (1.33)	9.00-9.81	19 (79.17)	9.13 (1.57)	8.50-9.75
	24. It has password management mechanisms.	33 (78.57)	9.05 (1.71)	8.53-9.56	19 (79.17)	9.04 (1.90)	8.28-9.80
	25. It states the terms and conditions of cloud services.	32 (76.19)	8.93 (2.23)	8.25-9.60	19 (79.17)	9.29 (1.08)	8.86-9.72
	26. The cloud services used have the relevant security measures.	36 (85.71)	9.40 (1.47)	8.96-9.85	21 (87.50)	9.29 (1.60)	8.65-9.93
	27. The authorization and authentication mechanisms protect users’ credentials and allow access to their data.	37 (88.10)	9.57 (1.02)	9.26-9.88	21 (87.50)	9.42 (1.21)	8.93-9.90
	28. It limits access to data that is only necessary for the user.	33 (78.57)	8.98 (2.25)	8.30-9.66	19 (79.17)	8.96 (2.10)	8.12-9.80
	29. It detects and identifies cybersecurity vulnerabilities, possible threats, and the risk of being exploited.	36 (85.71)	9.33 (1.76)	8.80-9.87	18 (75.00)	8.96 (2.16)	8.10-9.82
	30. It applies the appropriate security measures to cybersecurity vulnerabilities in the face of possible threats to reduce the risk of being exploited.	35 (83.33)	9.62 (0.82)	9.37-9.87	19 (79.17)	9.38 (0.92)	9.01-9.74
	31. It informs users of the possible risks associated with the app’s use of personal data.	—	—	—	20 (83.33)	9.25 (1.11)	8.80-9.70
**Appropriateness and suitability**							
	32. The benefits and advantages of using the app are explained.	31 (73.81)	8.95 (1.58)	8.48-9.43	18 (75.00)	9.08 (1.53)	8.47-9.70
	33. Experts have participated in the development of the app (for example, specialized professionals, health organizations, scientific societies, or specialized external organizations).	35 (83.33)	9.52 (1.02)	9.22-9.83	21 (87.50)	9.58 (0.72)	9.30-9.87
**Transparency and content**							
	34. It uses scientific evidence to guarantee the quality of the content.	36 (85.71)	9.60 (0.86)	9.34-9.85	20 (83.33)	9.46 (0.78)	9.15-9.77
	35. It is based on ethical principles and values.	39 (92.86)	9.71 (0.77)	9.48-9.95	22 (91.67)	9.75 (0.61)	9.51-9.99
**Safety**							
	36. The possible risks to users are identified.	36 (85.71)	9.45 (1.15)	9.10-9.80	20 (83.33)	9.46 (0.88)	9.10-9.81
	37. It ensures that there are no adverse effects.	—	—	—	18 (75.00)	8.92 (2.12)	8.07-9.77
	38. It complies with regulatory standards as a medical device.	—	—	—	22 (91.67)	9.46 (1.67)	8.79-10.13
	39. Users are warned when adverse events are identified so they can delete the app and avoid potential risks.	—	—	—	18 (75.00)	8.83 (1.93)	8.06-9.60
	40. Users are warned that the app is not meant to replace the services provided by a professional.	40 (95.24)	9.74 (0.63)	9.55-9.93	22 (91.67)	9.67 (0.64)	9.41-9.92
	41. It recommends always consulting a specialist in case of doubt.	—	—	—	22 (91.67)	9.33 (1.66)	8.67-10.00
	42. Potential risks for users caused by incorrect use and possible adverse effects are explained.	34 (80.95)	9.48 (0.92)	9.20-9.75	20 (83.33)	9.38 (1.17)	8.91-9.84
**Technical support and updates**							
	43. It gives a warning if updates can influence insensitive data (changes the use of the data or different data is collected).	32 (76.19)	8.90 (2.07)	8.28-9.53	19 (79.17)	9.17 (1.40)	8.60-9.73
	44. Every time an update of a third-party component is published, the change is inspected and the risk evaluated.	33 (78.57)	8.98 (1.81)	8.43-9.52	20 (83.33)	8.96 (1.97)	8.17-9.75
**Technology**							
	45. It works correctly. It does not fail during use (blocks, etc).	36 (85.71)	9.36 (1.23)	8.99-9.73	23 (95.83)	9.75 (0.53)	9.54-9.96
	46. Functions are correctly retrieved after context changes (switch to another app and return, etc), external interruptions (incoming calls or messages, etc), and switching off the terminal.	30 (71.43)	8.93 (1.50)	8.47-9.38	21 (87.50)	9.46 (0.83)	9.13-9.79
	47. It does not waste resources excessively: battery, central processing unit, memory, data, network, etc.	29 (69.05)	8.88 (1.48)	8.43-9.33	19 (79.17)	9.25 (1.11)	8.80-9.70
	48. It has a data recovery system in case of loss.	—	—	—	19 (79.17)	8.67 (2.28)	7.76-9.58

^a^These items were not available in round 1.

## Discussion

### Main Findings

The key finding from this study is that the MAG [28] is a valid tool to help guide the development of health-related mobile apps and assess their quality. The findings also indicate the items that are important to a health-related mobile app (the MAG is provided with this article; see Multimedia Appendix 2).

The data showed that 48 items on the MAG were considered to be of high importance (ie, they were rated with at least a 9 on a 0-10 numerical rating scale by at least 75% of the participants). Most of the items belonged to the categories *privacy* and *security*, thus showing that these are the issues that most concern stakeholders when assessing the quality of health mobile apps. In particular, the following items reached a consensus of 90%: *it clearly allows the user the option of nontransfer of data to third parties or for commercial purposes* (item 13); *it tells users when it accesses other resources on the mobile device, such as their accounts or their social network profiles* (item 17); *it takes measures to protect minors in accordance with current legislation* (item 18); *it complies with all current privacy laws* (item 22)*; it is based on ethical principles and values* (item 35); *it complies with regulatory standards as a medical device* (item 38); *users are warned that the app is not meant to replace the services provided by a professional* (item 40); *it recommends always consulting a specialist in case of doubt* (item 41); and *it works correctly, it does not fail during use (blocks, etc*; item 45).

Our work adds to previous proposals of quality guides or checklists by studying the validity of MAG, a comprehensive guide developed by Llorens-Vernet and Miró [28]. This guide was found to be a significant improvement on existing guides, as it had been developed with a comprehensive focus from a variety of sources (ie, research studies, recommendations from professional organizations, and standards governing the development of software for health or medical devices) and an international perspective (ie, resources used came from several regions worldwide). In addition, the guide was created to be of help to all stakeholders and not limited to a specific health problem.

### Future Research

Additional research is needed to establish the applicability of the MAG as a guide for health-related mobile app development. Future studies will have to test the MAG with real apps and check their functionality and usability among the different stakeholders who are interested in using it. Furthermore, studies to determine the relative importance of the items and the reliability and suitability of the guide in assessing mobile apps are also warranted. In this regard, a user version of the MAG will be developed to study the association between the quality of the user experience and the score in MAG. In the future, it is highly likely that additional items or criteria will be required to be able to look into the new functions and actions included in mobile apps. Thus, revised and updated versions of the MAG are to be expected.

### Limitations

The results of this study should be interpreted in the light of some limitations. The first of these is the representativeness of the participants. Although participants were an international sample of stakeholders, most of them were individuals living in Spain. We do not know if the results would have been the same with other experts. Nevertheless, for the most part, the group included individuals with extensive experience (in clinical work, research, and development), which suggests that their assessments are relevant and of good quality. Second, the number of participating experts changed from round 1 to round 2. However, this is quite normal and to be expected in all Delphi polls [23,28]. Although we cannot be certain that the results would have been the same had all participants in round 1 also responded to round 2, it is quite probable, as the differences between the rounds were minimal. Finally, the number of participants in each round was appropriate for the objectives (a minimum of 7 and maximum of 30 participants is recommended for studies like this; see [39,42]).

### Conclusions

Despite the limitations, the results of this study will help advance the field by providing developers, health care professionals, and end users with a valid guide (the MAG) for developing and identifying quality mHealth-related apps. The data shows that the stakeholders reached a consensus on 48 items distributed in 8 categories to establish them as the important criteria for health apps.

The MAG provides stakeholders with a valid tool for systematically reviewing health-related mobile apps. The MAG can be readily used to develop new apps by pointing to the general requirements that a mobile app ought to have if it is to be of high quality. Furthermore, the guide can help to rate existing apps and identify those that are of most interest on the basis of quality criteria. The apps that meet most of the criteria will give users the confidence that their objectives will be fulfilled. It can be used to provide a checklist for the evaluation and development of quality health apps.

## Appendix

Multimedia Appendix 1 Importance of the items in each round.

Multimedia Appendix 2 The Mobile App Development and Assessment Guide.

## Abbreviations

MAG: Mobile App Development and Assessment Guide
mHealth: mobile health
