# Supplementary material for: The Mobile App Development and Assessment Guide (MAG): Delphi-Based Validity Study
Source: JMIR Mhealth Uhealth. 2020 Jul 31;8(7):e17760. doi: 10.2196/17760 (PMC7428935; doi:10.2196/17760)
Supplement: Multimedia Appendix 1 [file mhealth_v8i7e17760_app1.docx]

**Importance of the items in each round**

|  |  | Round 1 (N=42) | | | Round 2 (N=24) | | |  |
| --- | --- | --- | --- | --- | --- | --- | --- | --- |
|  |  | Consensus, n (%) | Mean (SD) | CI | Consensus, n (%) | Mean (SD) | CI | P-value (between rounds) |
| Category | Item |  |  |  |  |  |  |  |
| **Usability** |  |  |  |  |  |  |  |  |
|  | 1. The app has been tested by potential users before being made available to the public. | 33 (78.57) | 9.14 (1.92) | 8.56 - 9.72 | 20 (83.33) | 9.21 (1.61) | 8.56 - 9.85 | .89 |
|  | 49. It has instructions or some kind of assistance for use. | 27 (64.29) | 8.62 (2.19) | 7.96 - 9.28 | 12 (50.00) | 8.46 (1.67) | 7.79 - 9.13 | .76 |
|  | 2. It is easy to use (that is, navigation is intuitive). | 39 (92.86) | 9.67 (0.61) | 9.48 - 9.85 | 21 (87.50) | 9.50 (0.72) | 9.21 - 9.79 | .32 |
|  | 50. It follows the recommendations, patterns and directives in the official manuals of the different operating systems (Android, iOS, etc.). | 24 (57.14) | 8.40 (1.80) | 7.86 - 8.95 | 12 (50.00) | 8.42 (1.56) | 7.79 - 9.04 | .98 |
|  | 51. The interface is suitable for different types of mobile devices. | - | - | - | 16 (66.67) | 9.04 (1.30) | 8.52 - 9.56 | - |
|  | 52. The interface design follows the same pattern. That is, all graphic elements (typography, icons, buttons, color contrast, etc.) have a consistent appearance. | 27 (64.29) | 8.64 (1.71) | 8.13 - 9.16 | 14 (58.33) | 8.46 (1.72) | 7.77 - 9.15 | .68 |
|  | 53. The function of each element (navigation menu, lists, photo gallery, etc.) is clearly identified. | 30 (71.43) | 8.93 (1.40) | 8.50 - 9.35 | 14 (58.33) | 8.71 (1.57) | 8.08 - 9.34 | .56 |
|  | 3. Functionality is adapted to the purpose of the application. | 40 (95.24) | 9.74 (0.63) | 9.55 - 9.93 | 21 (87.50) | 9.42 (0.93) | 9.05 - 9.79 | .10 |
|  | 4. Functionality is adjusted according to the profile and needs of the targeted user. | - | - | - | 19 (79.17) | 9.08 (0.93) | 8.71 - 9.45 | - |
|  | 5. Access is adapted for people with disabilities. | - | - | - | 19 (79.17) | 9.00 (1.41) | 8.43 - 9.57 | - |
|  | 6. It complies with regulatory accessibility standards. | - | - | - | 18 (75.00) | 9.17 (1.13) | 8.71 - 9.62 | - |
|  | 7. The language used makes it accessible to any user. | - | - | - | 19 (79.17) | 9.04 (1.55) | 8.42 - 9.66 | - |
|  | 54. The app and its functions are designed in a way that facilitates the work of administrators. | - | - | - | 9 (37.50) | 8.13 (1.19) | 7.65 - 8.60 | - |
|  | 55. The information in the application is accessed in the shortest possible time. | 27 (64.29) | 8.83 (1.41) | 8.41 - 9.26 | 14 (58.33) | 8.75 (1.22) | 8.26 - 9.24 | .81 |
|  | 8. All users have access to all resources regardless of their capabilities. | 28 (66.67) | 8.60 (1.67) | 8.09 - 9.10 | 18 (75.00) | 9.08 (1.06) | 8.66 - 9.51 | .20 |
|  | 56. The app can be consulted in more than one language. | 17 (40.48) | 7.29 (2.66) | 6.48 - 8.09 | 10 (41.67) | 7.63 (2.14) | 6.77 - 8.48 | .60 |
|  | 57. All languages adapt appropriately to the content interface. | 23 (54.76) | 8.02 (2.35) | 7.31 - 8.74 | 11 (45.83) | 7.88 (2.38) | 6.92 - 8.83 | .81 |
|  | 58. It allows users to create their own avatars. | - | - | - | 6 (25.00) | 5.75 (3.08) | 4.52 - 6.98 | - |
| **Privacy** |  |  |  |  |  |  |  |  |
|  | 9. The app gives information about the terms and conditions of purchases in the application. | 35 (83.33) | 9.29 (1.90) | 8.71 - 9.86 | 21 (87.50) | 9.50 (1.18) | 9.03 - 9.97 | .62 |
|  | 10. It must only ask for user data that is essential for the application to operate. | 34 (80.95) | 9.26 (1.53) | 8.80 - 9.72 | 18 (75.00) | 8.92 (2.04) | 8.10 - 9.73 | .44 |
|  | 59. It provides functionalities that do not collect any personal data. | - | - | - | 11 (45.83) | 8.04 (1.92) | 7.27 - 8.81 | - |
|  | 60. It gives information about the kind of user data to be collected and the reason. | 30 (71.43) | 9.05 (1.64) | 8.55 - 9.54 | 17 (70.83) | 9.21 (1.06) | 8.78 - 9.63 | .67 |
|  | 11. It gives information about access policies and data processing, and ensures the right of access to recorded information. | 34 (80.95) | 9.02 (2.25) | 8.34 - 9.70 | 18 (75.00) | 9.38 (0.97) | 8.99 - 9.76 | .47 |
|  | 61. It describes the maintenance policy and the data erasure procedure. | 28 (66.67) | 8.33 (2.80) | 7.49 - 9.18 | 16 (66.67) | 8.71 (1.78) | 8.00 - 9.42 | .57 |
|  | 12. It gives information about possible commercial agreements with third parties. | 32 (76.19) | 8.79 (2.54) | 8.02 - 9.55 | 20 (83.33) | 9.17 (2.12) | 8.32 - 10.01 | .54 |
|  | 13. It clearly allows the user the option of non-transfer of data to third parties or for commercial purposes. | - | - | - | 23 (95.83) | 9.71 (0.55) | 9.49 - 9.93 | - |
|  | 14. It guarantees the privacy of the information recorded. | 39 (92.86) | 9.71 (0.77) | 9.48 - 9.95 | 20 (83.33) | 9.46 (1.10) | 9.02 - 9.90 | .27 |
|  | 15. It requires users to give their express consent. | 36 (85.71) | 9.12 (2.19) | 8.46 - 9.78 | 19 (79.17) | 9.38 (1.01) | 8.97 - 9.78 | .59 |
|  | 16. It warns of the risks of using the application. | 36 (85.71) | 9.33 (1.86) | 8.77 - 9.89 | 19 (79.17) | 9.25 (1.19) | 8.77 - 9.73 | .84 |
|  | 17. It tells users when it accesses other resources on the mobile device such as their accounts or their social network profiles. | 36 (85.71) | 9.33 (1.76) | 8.80 - 9.87 | 22 (91.67) | 9.71 (0.75) | 9.41 - 10.01 | .33 |
|  | 18. It takes measures to protect minors in accordance with current legislation. | 38 (90.48) | 9.43 (1.74) | 8.90 - 9.96 | 23 (95.83) | 9.79 (0.51) | 9.59 - 10.00 | .32 |
|  | 19. Confidential user data is protected and anonymized, and there is a privacy mechanism so that users can control their data. | 38 (90.48) | 9.60 (1.06) | 9.27 - 9.92 | 21 (87.50) | 9.46 (1.18) | 8.99 - 9.93 | .63 |
|  | 20. It offers to erase the data when the service is finished. | - | - | - | 19 (79.17) | 9.04 (1.68) | 8.37 - 9.71 | - |
|  | 21. It gives information about privacy policies in a simple and understandable way. | - | - | - | 20 (83.33) | 9.33 (1.09) | 8.90 - 9.77 | - |
|  | 62. The application can only be accessed by means of a prescription from a professional. | - | - | - | 2 (8.33) | 4.96 (3.04) | 3.74 - 6.18 | - |
|  | 22. It complies with all current privacy laws. | - | - | - | 22 (91.67) | 9.54 (1.28) | 9.03 - 10.06 | - |
|  | 63. It offers the possibility of sharing the data through different means (e.g., social networks). | - | - | - | 6 (25.00) | 7.04 (2.56) | 6.02 - 8.07 | - |
| **Security** |  |  |  |  |  |  |  |  |
|  | 23. The app has encryption mechanisms for storing, collecting and exchanging information. | 35 (83.33) | 9.40 (1.33) | 9.00 - 9.81 | 19 (79.17) | 9.13 (1.57) | 8.50 - 9.75 | .44 |
|  | 24. It has password management mechanisms. | 33 (78.57) | 9.05 (1.71) | 8.53 - 9.56 | 19 (79.17) | 9.04 (1.90) | 8.28 - 9.80 | .99 |
|  | 25. It states the terms and conditions of cloud services. | 32 (76.19) | 8.93 (2.23) | 8.25 - 9.60 | 19 (79.17) | 9.29 (1.08) | 8.86 - 9.72 | .46 |
|  | 26. The cloud services used have the relevant security measures. | 36 (85.71) | 9.40 (1.47) | 8.96 - 9.85 | 21 (87.50) | 9.29 (1.60) | 8.65 - 9.93 | .77 |
|  | 64. It uses the cloud of a specialized company to outsource cybersecurity. | - | - | - | 5 (20.83) | 6.71 (2.29) | 5.79 - 7.63 | - |
|  | 27. The authorization and authentication mechanisms protect users’ credentials and allow access to their data. | 37 (88.10) | 9.57 (1.02) | 9.26 - 9.88 | 21 (87.50) | 9.42 (1.21) | 8.93 - 9.90 | .58 |
|  | 28. It limits access to data that is only necessary for the user. | 33 (78.57) | 8.98 (2.25) | 8.30 - 9.66 | 19 (79.17) | 8.96 (2.10) | 8.12 - 9.80 | .97 |
|  | 29. It detects and identifies cybersecurity vulnerabilities, possible threats and the risk of being exploited. | 36 (85.71) | 9.33 (1.76) | 8.80 - 9.87 | 18 (75.00) | 8.96 (2.16) | 8.10 - 9.82 | .45 |
|  | 30. It applies the appropriate security measures to cybersecurity vulnerabilities in the face of possible threats, in order to reduce the risk of being exploited. | 35 (83.33) | 9.62 (0.82) | 9.37 - 9.87 | 19 (79.17) | 9.38 (0.92) | 9.01 - 9.74 | .27 |
|  | 65. It does not require complex passwords | - | - | - | 6 (25.00) | 6.38 (2.93) | 5.20 - 7.55 | - |
|  | 66. It informs users about the security measures implemented in an understandable way. | - | - | - | 14 (58.33) | 8.71 (1.68) | 8.04 - 9.38 | - |
|  | 67. It uses the latest available technologies to protect data in the best possible way. | - | - | - | 17 (70.83) | 9.04 (1.08) | 8.61 - 9.47 | - |
|  | 31. It informs users of the possible risks associated with the application’s use of personal data. | - | - | - | 20 (83.33) | 9.25 (1.11) | 8.80 - 9.70 | - |
| **Appropriateness and suitability** |  |  |  |  |  |  |  |  |
|  | 68. The audience the app is intended for is clearly stated. | 28 (66.67) | 8.71 (1.74) | 8.19 - 9.24 | 15 (62.50) | 8.67 (1.69) | 7.99 - 9.34 | .91 |
|  | 32. The benefits and advantages of using the app are explained. | 31 (73.81) | 8.95 (1.58) | 8.48 - 9.43 | 18 (75.00) | 9.08 (1.53) | 8.47 - 9.70 | .74 |
|  | 69. It has been validated scientifically and shows the results of the validation. | 35 (83.33) | 9.52 (1.02) | 9.22 - 9.83 | 15 (62.50) | 8.71 (2.14) | 7.85 - 9.56 | .04 |
|  | 33. Experts have participated in the development of the app (for example, specialized professionals, health organizations, scientific societies or specialized external organizations). | 35 (83.33) | 9.52 (1.02) | 9.22 - 9.83 | 21 (87.50) | 9.58 (0.72) | 9.30 - 9.87 | .80 |
|  | 70. Patients have been involved in its development. | - | - | - | 16 (66.67) | 8.21 (2.80) | 7.09 - 9.33 | - |
| **Transparency and content** |  |  |  |  |  |  |  |  |
|  | 71. The app identifies the authors of the content and their professional qualifications. | 25 (59.52) | 8.55 (2.04) | 7.93 - 9.16 | 16 (66.67) | 8.63 (1.86) | 7.88 - 9.37 | .88 |
|  | 72. It identifies the institution that supports it. | - | - | - | 17 (70.83) | 8.67 (2.14) | 7.81 - 9.52 | - |
|  | 73. It gives transparent information about the owner’s identity and location over time. | 27 (64.29) | 8.62 (1.85) | 8.06 - 9.18 | 16 (66.67) | 8.58 (1.95) | 7.80 - 9.37 | .94 |
|  | 74. It gives information about its sources of funding, promotion and sponsorship, as well as possible conflicts of interests. | 21 (50.00) | 7.52 (2.80) | 6.68 - 8.37 | 14 (58.33) | 7.75 (2.66) | 6.69 - 8.81 | .75 |
|  | 75. Any third parties and/or organizations that have contributed to the app’s development are clearly identified. | 19 (45.24) | 7.26 (3.00) | 6.36 - 8.17 | 13 (54.17) | 7.46 (3.12) | 6.21 - 8.71 | .80 |
|  | 34. It uses scientific evidence to guarantee the quality of the content. | 36 (85.71) | 9.60 (0.86) | 9.34 - 9.85 | 20 (83.33) | 9.46 (0.78) | 9.15 - 9.77 | .52 |
|  | 35. It is based on ethical principles and values. | 39 (92.86) | 9.71 (0.77) | 9.48 - 9.95 | 22 (91.67) | 9.75 (0.61) | 9.51 - 9.99 | .85 |
|  | 76. The sources of the information are cited. | 33 (78.57) | 9.07 (1.40) | 8.65 - 9.50 | 16 (66.67) | 8.50 (2.30) | 7.58 - 9.42 | .21 |
|  | 77. Concise information is given about the procedure used to select the content. | 18 (42.86) | 7.74 (2.29) | 7.05 - 8.43 | 10 (41.67) | 7.92 (2.08) | 7.08 - 8.75 | .75 |
|  | 78. It demonstrates the involvement of patients in the development of the application. | - | - | - | 12 (50.00) | 6.79 (3.65) | 5.33 - 8.25 |  |
|  | 79. It provides additional content such as healthy lifestyle advice and recommendations for patients. | - | - | - | 12 (50.00) | 7.33 (3.20) | 6.05 - 8.61 |  |
| **Safety** |  |  |  |  |  |  |  |  |
|  | 36. The possible risks to users are identified. | 36 (85.71) | 9.45 (1.15) | 9.10 - 9.80 | 20 (83.33) | 9.46 (0.88) | 9.10 - 9.81 | .98 |
|  | 37. It ensures that there are no adverse effects. | - | - | - | 18 (75.00) | 8.92 (2.12) | 8.07 - 9.77 | - |
|  | 38. It complies with regulatory standards as a medical device. | - | - | - | 22 (91.67) | 9.46 (1.67) | 8.79 - 10.13 | - |
|  | 39. Users are warned when adverse events are identified so they can delete the application and avoid potential risks. | - | - | - | 18 (75.00) | 8.83 (1.93) | 8.06 - 9.60 | - |
|  | 40. Users are warned that the app is not meant to replace the services provided by a professional. | 40 (95.24) | 9.74 (0.63) | 9.55 - 9.93 | 22 (91.67) | 9.67 (0.64) | 9.41 - 9.92 | .66 |
|  | 41. It recommends always consulting a specialist in case of doubt. | - | - | - | 22 (91.67) | 9.33 (1.66) | 8.67 - 10.00 | - |
|  | 42. Potential risks for users caused by incorrect usage and/or possible adverse effects are explained. | 34 (80.95) | 9.48 (0.92) | 9.20 - 9.75 | 20 (83.33) | 9.38 (1.17) | 8.91 - 9.84 | .70 |
|  | 80. It gives the option of contacting a doctor. | - | - | - | 10 (41.67) | 7.17 (3.23) | 5.88 - 8.46 | - |
| **Technical support and updates** |  |  |  |  |  |  |  |  |
|  | 81. It gives a warning if updates modify or affect how the app functions. | 25 (59.52) | 8.76 (1.72) | 8.24 - 9.28 | 14 (58.33) | 8.67 (1.88) | 7.91 - 9.42 | .84 |
|  | 43. It gives a warning if updates can influence insensitive data (changes the use of the data or different data is collected). | 32 (76.19) | 8.90 (2.07) | 8.28 - 9.53 | 19 (79.17) | 9.17 (1.40) | 8.60 - 9.73 | .58 |
|  | 82. Frequent security updates are guaranteed. | 28 (66.67) | 8.79 (1.72) | 8.27 - 9.31 | 15 (62.50) | 8.50 (1.87) | 7.75 - 9.25 | .53 |
|  | 44. Every time an update of a third-party component is published, the change is inspected and the risk evaluated. | 33 (78.57) | 8.98 (1.81) | 8.43 - 9.52 | 20 (83.33) | 8.96 (1.97) | 8.17 - 9.75 | .97 |
|  | 83. The frequency with which the content of the app is revised or updated is shown. | 19 (45.24) | 7.86 (2.18) | 7.20 - 8.52 | 13 (54.17) | 8.38 (2.10) | 7.53 - 9.22 | .35 |
|  | 84. Users have support mechanisms (e-mail, phone, contact form) for resolving questions, problems or issues related to the health content and technical support. | 28 (66.67) | 8.98 (1.51) | 8.52 - 9.43 | 15 (62.50) | 8.67 (1.66) | 8.00 - 9.33 | .44 |
|  | 85. Users receive responses to their queries within 24h on weekdays and 48h on holidays, when needed. | - | - | - | 11 (45.83) | 7.75 (2.19) | 6.87 - 8.63 | - |
|  | 86. It has a policy of updates and long-term maintenance. | - | - | - | 13 (54.17) | 8.21 (1.84) | 7.47 - 8.94 | - |
|  | 87. It keeps old mobile devices updated without compromising the latest advantages available for more modern mobile devices. | - | - | - | 13 (54.17) | 8.00 (2.06) | 7.17 - 8.83 | - |
| **Technology** |  |  |  |  |  |  |  |  |
|  | 45. It works correctly. It does not fail during use (blocks, etc.). | 36 (85.71) | 9.36 (1.23) | 8.99 - 9.73 | 23 (95.83) | 9.75 (0.53) | 9.54 - 9.96 | .14 |
|  | 46. Functions are correctly retrieved after context changes (switch to another app and return, etc.), external interruptions (incoming calls or messages, etc.) and switching off the terminal. | 30 (71.43) | 8.93 (1.50) | 8.47 - 9.38 | 21 (87.50) | 9.46 (0.83) | 9.13 - 9.79 | .12 |
|  | 47. It does not waste resources excessively: battery, CPU, memory, data, network, etc. | 29 (69.05) | 8.88 (1.48) | 8.43 - 9.33 | 19 (79.17) | 9.25 (1.11) | 8.80 - 9.70 | .29 |
|  | 88. It informs the user about data consumption when in use. | - | - | - | 12 (50.00) | 8.04 (2.24) | 7.15 - 8.94 | - |
|  | 89. It can work in flight mode, and deal with network delays and any loss of connection. | 17 (40.48) | 7.90 (1.79) | 7.36 - 8.45 | 15 (62.50) | 8.17 (2.26) | 7.26 - 9.07 | .61 |
|  | 90. It allows the user to choose what type of connection to use: WiFi or mobile data. | - | - | - | 13 (54.17) | 8.08 (2.24) | 7.19 - 8.98 | - |
|  | 91. It supports multiple versions of data structures or formats and different versions of the operating system. | 29 (69.05) | 8.79 (1.88) | 8.22 - 9.35 | 13 (54.17) | 8.25 (2.45) | 7.27 - 9.23 | .32 |
|  | 92. It can regulate the use of sounds, brightness, access, interchangeability, etc. | - | - | - | 13 (54.17) | 8.25 (2.17) | 7.38 - 9.12 | - |
|  | 48. It has a data recovery system in case of loss. | - | - | - | 19 (79.17) | 8.67 (2.28) | 7.76 - 9.58 | - |
