# Supplementary material for: The Mobile App Development and Assessment Guide (MAG): Delphi-Based Validity Study
Source: JMIR Mhealth Uhealth. 2020 Jul 31;8(7):e17760. doi: 10.2196/17760 (PMC7428935; doi:10.2196/17760)
Supplement: Multimedia Appendix 2 [file mhealth_v8i7e17760_app2.docx]

**The Mobile App Development and Assessment Guide (MAG)**

**Usability**

| **#** | **Criterion** | **Check** |
| --- | --- | --- |
|  |  |  |
| 1 | The app has been tested by potential users before being made available to the public. |  |
| 2 | It is easy to use (that is, navigation is intuitive). |  |
| 3 | Functionality is adapted to the purpose of the application. |  |
| 4 | Functionality is adjusted according to the profile and needs of the targeted user. |  |
| 5 | Access is adapted for people with disabilities. |  |
| 6 | It complies with regulatory accessibility standards. |  |
| 7 | The language used makes it accessible to any user. |  |
| 8 | All users have access to all resources regardless of their capabilities. |  |

**Privacy**

| **#** | **Criterion** | **Check** |
| --- | --- | --- |
|  |  |  |
| 9 | The app gives information about the terms and conditions of purchases in the application. |  |
| 10 | It must only ask for user data that is essential for the application to operate. |  |
| 11 | It gives information about access policies and data processing, and ensures the right of access to recorded information. |  |
| 12 | It gives information about possible commercial agreements with third parties. |  |
| 13 | It clearly allows the user the option of non-transfer of data to third parties or for commercial purposes. |  |
| 14 | It guarantees the privacy of the information recorded. |  |
| 15 | It requires users to give their express consent. |  |
| 16 | It warns of the risks of using the application. |  |
| 17 | It tells users when it accesses other resources on the mobile device such as their accounts or their social network profiles. |  |
| 18 | It takes measures to protect minors in accordance with current legislation. |  |
| 19 | Confidential user data is protected and anonymized, and there is a privacy mechanism so that users can control their data. |  |
| 20 | It offers to erase the data when the service is finished. |  |
| 21 | It gives information about privacy policies in a simple and understandable way. |  |
| 22 | It complies with all current privacy laws. |  |

**Security**

| **#** | **Criterion** | **Check** |
| --- | --- | --- |
|  |  |  |
| 23 | The app has encryption mechanisms for storing, collecting and exchanging information. |  |
| 24 | It has password management mechanisms. |  |
| 25 | It states the terms and conditions of cloud services. |  |
| 26 | The cloud services used have the relevant security measures. |  |
| 27 | The authorization and authentication mechanisms protect users’ credentials and allow access to their data. |  |
| 28 | It limits access to data that is only necessary for the user. |  |
| 29 | It detects and identifies cybersecurity vulnerabilities, possible threats and the risk of being exploited. |  |
| 30 | It applies the appropriate security measures to cybersecurity vulnerabilities in the face of possible threats, in order to reduce the risk of being exploited. |  |
| 31 | It informs users of the possible risks associated with the application’s use of personal data. |  |

**Appropriateness and suitability**

| **#** | **Criterion** | **Check** |
| --- | --- | --- |
|  |  |  |
| 32 | The benefits and advantages of using the app are explained. |  |
| 33 | Experts have participated in the development of the app (for example, specialized professionals, health organizations, scientific societies or specialized external organizations). |  |

**Transparency and content**

| **#** | **Criterion** | **Check** |
| --- | --- | --- |
|  |  |  |
| 34 | It uses scientific evidence to guarantee the quality of the content. |  |
| 35 | It is based on ethical principles and values. |  |

**Safety**

| **#** | **Criterion** | **Check** |
| --- | --- | --- |
|  |  |  |
| 36 | The possible risks to users are identified. |  |
| 37 | It ensures that there are no adverse effects. |  |
| 38 | It complies with regulatory standards as a medical device. |  |
| 39 | Users are warned when adverse events are identified so they can delete the application and avoid potential risks. |  |
| 40 | Users are warned that the app is not meant to replace the services provided by a professional. |  |
| 41 | It recommends always consulting a specialist in case of doubt. |  |
| 42 | Potential risks for users caused by incorrect usage and/or possible adverse effects are explained. |  |

**Technical support and updates**

| **#** | **Criterion** | **Check** |
| --- | --- | --- |
|  |  |  |
| 43 | It gives a warning if updates can influence insensitive data (changes the use of the data or different data is collected). |  |
| 44 | Every time an update of a third-party component is published, the change is inspected and the risk evaluated. |  |

**Technology**

| **#** | **Criterion** | **Check** |
| --- | --- | --- |
|  |  |  |
| 45 | It works correctly. It does not fail during use (blocks, etc.). |  |
| 46 | Functions are correctly retrieved after context changes (switch to another app and return, etc.), external interruptions (incoming calls or messages, etc.) and switching off the terminal. |  |
| 47 | It does not waste resources excessively: battery, CPU, memory, data, network, etc. |  |
| 48 | It has a data recovery system in case of loss. |  |
